# GSHR, a Web-Based Platform Provides Gene Set-Level Analyses of Hormone Responses in *Arabidopsis*

**DOI:** 10.3389/fpls.2018.00023

**Published:** 2018-01-24

**Authors:** Xiaojuan Ran, Jian Liu, Meifang Qi, Yuejun Wang, Jingfei Cheng, Yijing Zhang

**Affiliations:** ^1^National Key Laboratory of Plant Molecular Genetics, CAS Center for Excellence in Molecular Plant Sciences, Institute of Plant Physiology and Ecology, Shanghai Institutes for Biological Sciences, Chinese Academy of Sciences, Shanghai, China; ^2^University of Chinese Academy of Sciences, Beijing, China

**Keywords:** GSHR, *Arabidopsis thaliana*, web-based platform, hormone, transcriptome, gene set-level, cross-study, cross-platform

## Abstract

Phytohormones regulate diverse aspects of plant growth and environmental responses. Recent high-throughput technologies have promoted a more comprehensive profiling of genes regulated by different hormones. However, these omics data generally result in large gene lists that make it challenging to interpret the data and extract insights into biological significance. With the rapid accumulation of theses large-scale experiments, especially the transcriptomic data available in public databases, a means of using this information to explore the transcriptional networks is needed. Different platforms have different architectures and designs, and even similar studies using the same platform may obtain data with large variances because of the highly dynamic and flexible effects of plant hormones; this makes it difficult to make comparisons across different studies and platforms. Here, we present a web server providing gene set-level analyses of *Arabidopsis thaliana* hormone responses. GSHR collected 333 RNA-seq and 1,205 microarray datasets from the Gene Expression Omnibus, characterizing transcriptomic changes in *Arabidopsis* in response to phytohormones including abscisic acid, auxin, brassinosteroids, cytokinins, ethylene, gibberellins, jasmonic acid, salicylic acid, and strigolactones. These data were further processed and organized into 1,368 gene sets regulated by different hormones or hormone-related factors. By comparing input gene lists to these gene sets, GSHR helped to identify gene sets from the input gene list regulated by different phytohormones or related factors. Together, GSHR links prior information regarding transcriptomic changes induced by hormones and related factors to newly generated data and facilities cross-study and cross-platform comparisons; this helps facilitate the mining of biologically significant information from large-scale datasets. The GSHR is freely available at http://bioinfo.sibs.ac.cn/GSHR/.

## Introduction

Phytohormones are typically small endogenous compounds regulating every aspect of plant life, from plant growth and development to responses to environmental changes (Santner and Estelle, 2009; Wolters and Jurgens, 2009; Scheres and van der Putten, 2017). Specific phytohormones are synthesized in response to distinct developmental or environmental cues, which are perceived by specific receptors and further activate cascades of signaling pathways, leading to remarkable biochemical and physiological changes (Santner and Estelle, 2009; Wolters and Jurgens, 2009; Scheres and van der Putten, 2017). The major hormones were traditionally classified into two categories, those mainly regulating developmental processes including auxin, brassinosteroid (BR), cytokinin (CK), and gibberellic acid (GA) and strigolactones, and those regulating stress responses, including abscisic acid (ABA), ethylene (ET), jasmonic acid (JA), and salicylic acid (SA). Over the past decades, wide-spread crosstalk was revealed among various phytohormone pathways in response to specific developmental or environmental changes (Depuydt and Hardtke, 2011; Robert-Seilaniantz et al., 2011; Murphy, 2015). Typical examples include the antagonistic role of GA and ABA in controlling seed dormancy (del Carmen Rodríguez-Gacio et al., 2009; Ye and Zhang, 2012; Shu et al., 2016), the interaction between auxin and CK in regulating meristem development (Kuderova and Hejatko, 2009; Su et al., 2011; Azizi et al., 2015). The crosstalk happens on multiple layers of regulation, among which transcriptional control is one major component.

Genetic studies identified various specific transcription factors mediating phytohormone signaling pathways; mutations in these factors resulted in plants that became more sensitive or insensitive to hormone treatments (Moore et al., 2011; Eckardt, 2015; Tsuda and Somssich, 2015). The interaction among factors regulating hormone signaling and factors controlling growth and responses mediates the crosstalk of different hormones in particular conditions. For example, the antagonistic interaction between auxin and CK in controlling root meristem development is achieved via the regulatory circuit between auxin signaling factors SHORT HYPOCOTYL 2 (SHY2/IAA3) and CK signaling regulator ARABIDOPSIS RESPONSE REGULATOR1 (ARR1) (Kuderova and Hejatko, 2009; Su et al., 2011). In another example, the central component regulating dark morphogenesis phytochrome interacting factor (PIF4) actively participated in auxin and BR-mediated pathways (Oh et al., 2012, 2014; Wang et al., 2014; Chaiwanon et al., 2016; Franciosini et al., 2017). To systematically characterize the crosstalk is non-trivial due to the complicated signaling network. The transcription of 100–1000 of genes display significant changes in response to various hormone treatments, and to dissect their common and unique targets is the pre-requisite for a comprehensive understanding of the crosstalk. During the past decades, high-throughput technologies have promoted systematic characterization of the signaling pathways triggered by various phytohormones and related factors. For example, microarray and RNA-seq experiments were performed to profile the transcriptomic changes triggered by phytohormones (Paponov et al., 2008; Garg et al., 2012) while chromatin immunoprecipitation followed by sequencing (ChIP-seq) studies have facilitated the identification of genes regulated by hormone-related factors (Oh et al., 2012, 2014; Song et al., 2016; Zhu et al., 2016). It was revealed that both phytohormones and related factors have wide-spread targets, and much research on phytohormones now involves investigating how plant hormones interact with each other and with related factors to orchestrate plant transcriptomic network (Depuydt and Hardtke, 2011; Eckardt, 2015). Accordingly, it is important to compare the high-throughput data obtained from different studies and from different platforms. However, such comparison is a challenging task for a number of reasons. Firstly, different platforms have different architectures and designs, and the results are not directly comparable (Barnes et al., 2005; Nookaew et al., 2012). For example, the probe intensity obtained from the Affymetrix platform cannot directly be compared to that from the Agilent platform due to different technical procedures such as the probe design, chip fabrication and data analysis (Sah et al., 2010; Del Vescovo et al., 2013); the results obtained from ChIP-seq experiments are not directly comparable to those from RNA-seq studies. Secondly, given the dynamic and flexible effects of phytohormones, similar experiments from different labs or even from different repeats in the same lab may generate data with large variances that are not readily comparable (Lee and Park, 2010; Rivas-San Vicente and Plasencia, 2011). To obtain more robust results, researchers proposed the idea of module-level analysis; this derives its power by focusing on gene sets sharing common biological functions or expression behavior. The basic procedure is to pre-define specific gene sets based on the comprehensive collection of information from dry-lab and wet-lab experiments. For any given gene list, the potential function of these genes could be deduced based on large amounts of prior information. Typical examples include Gene Set Enrichment Analysis (GSEA) and the Database for Annotation, Visualization and Integrated Discovery (DAVID), which are mostly for animals (Dennis et al., 2003; Subramanian et al., 2005), PlantGSEA for plants (Yi et al., 2013), and Comprehensive Annotation of Rice Omics-data (CARMO) for rice (Wang et al., 2015).

Given the vast amount of hormone-related transcriptomic data accumulated in public databases, the ability to make full use of available high-throughput data is of great significance. Here, we present gene set-level analyses of hormone responses in *Arabidopsis* (GSHR), a web server that provides analyses of phytohormone responses based on the integration of hormone-responsive gene modules. GSHR collected 333 RNA-seq and 1,205 microarray samples from the Gene Expression Omnibus that were organized into 1,368 gene lists regulated by different hormones or hormone-related factors. This gene list-level analysis helps reach more reliable and robust conclusions about which genes under study were affected by what types of plant hormones or related factors; three examples were used to illustrate the power of GSHR in data mining of hormone related information.

## Materials and Methods

### Data Collection

1,538 phytohormone-related transcriptomic datasets in *Arabidopsis thaliana* were collected from Gene Expression Omnibus (GEO^1^), including 73 studies with 1,205 datasets generated using microarray platforms (Affymetrix or Agilent), and 21 studies with 333 datasets generated by RNA-seq based on Illumina sequencing platform (Supplementary Table 1). All relevant comparisons between datasets of the same study were performed, then we defined the up-regulated and down-regulated genes in one pair comparison respectively as a gene set, finally generating 1,368 gene sets. We extracted the hormones or the transcription factors, mutants, or genes involved in hormone signaling used in the comparison as hormone or hormone-related factors of the gene set.

### Process of RNA-Seq Data

RNA-seq reads with MAPQ < 20 were filtered followed by adapter trimming using trim galore^2^ with default settings. The cleaned reads were mapped to Col-0 genome (Araport11) using STAR (Dobin et al., 2013), and the number of reads in each gene was counted. Differentially expressed genes were calculated via DEseq (Anders and Huber, 2010) with the combined criteria: |log_2_(fold change)| >1, p value<0.05.

### Process of Microarray Data

Microarray data from Affymetrix platform were pre-processed with Bioconductor package affy (Gautier et al., 2004; Gentleman et al., 2004), and gcrma function was used for background correction and normalization (Wu and Irizarry, 2004). Microarray data from Agilent platform were normalized using quantile normalization (Bolstad et al., 2003). In the case of genes with multiple probes, the probe with the largest expression intensity was kept. For both platforms, linear model in Bioconductor package limma (Smyth, 2005) was applied for identifying differentially expressed genes with combined criteria:|log_2_ (fold change)| >1 and p value<0.01. Comparisons resulting in >10 differentially expressed genes were kept.

### Enrichment Analysis

Fisher’s exact test (Routledge, 2005) was used for the enrichment analysis, which is displayed as follows:

$$\text{p}=\frac{\left(\begin{array}{c} \text{n}\\ \text{k} \end{array})(\begin{array}{c} \text{N}-\text{n}\\ \text{K}-\text{k} \end{array}\right)}{\left(\begin{array}{c} \text{N}\\ \text{K} \end{array}\right)}$$

where N is the total number of genes as background. Different N is used for different analysis. For comparison of gene sets from transcriptomic data, there are 24,327 genes in total; and the numbers for KEGG, gene ontology and InterPro domain analysis are 4,797, 30,468 and 15,816, respectively. n is the number of input genes. K is the total number of genes in one gene set and k strands for the overlapped genes between input and the pre-defined gene sets. For multiple testing correction, the adjusted P values calculated by FDR, Bonferroni correction and Benjamini and Hochberg method were provided.

The enriched fold change is the fraction of input genes involved in the given gene set divided by the fraction of all annotated genes involved in the gene set. This is commonly used for functional term enrichment analysis, e.g., DAVID bioinformatics Resources: https://david.ncifcrf.gov/term2term.jsp. The calculation formula is:

$$\text{Fold Change}=\frac{\text{n}/\text{k}}{\text{N}/\text{K}}$$

The values of N, n, k and K are descripted as above and presented in the download file, corresponding to PopTotal, ListTotal, Count and PopHits columns, respectively.

### Construction of Co-expression Network

We selected 22,090 genes present in both microarray and RNA-seq datasets for construction of co-expression network. log_2_(fold change) of each gene from all pair-wise comparisons were collected, and the function rcorr in R package Hmisc (Harrell, 2017) was used to calculated the Pearson correlation coefficients and *p*-values between a pair of genes. The genes pairs with coefficients above 0.70 were used to construct the co-expression network.

### Web Server Implementation

The GSHR was constructed on Apache HTTP Server based on Linux system, MySQL was used for storage and operation of the database, the web interface is supported by PHP and JavaScript scripts. We used Python for statistical analysis and data processing, cluster analysis was performed in R. The JavaScript Library d3.js was used for co-expression network visualization. All scripts are available upon request.

## Results

### Structure of GSHR

**FIGURE 1:**
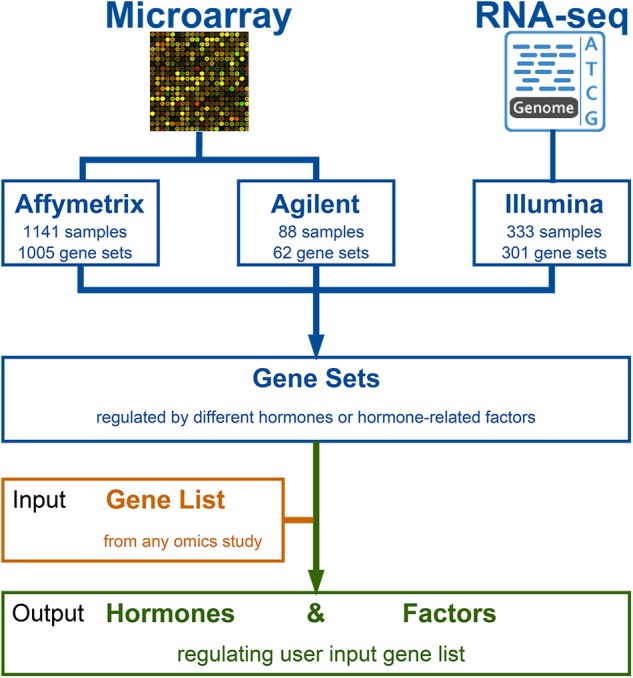
The framework and content of GSHR.

**FIGURE 2:**
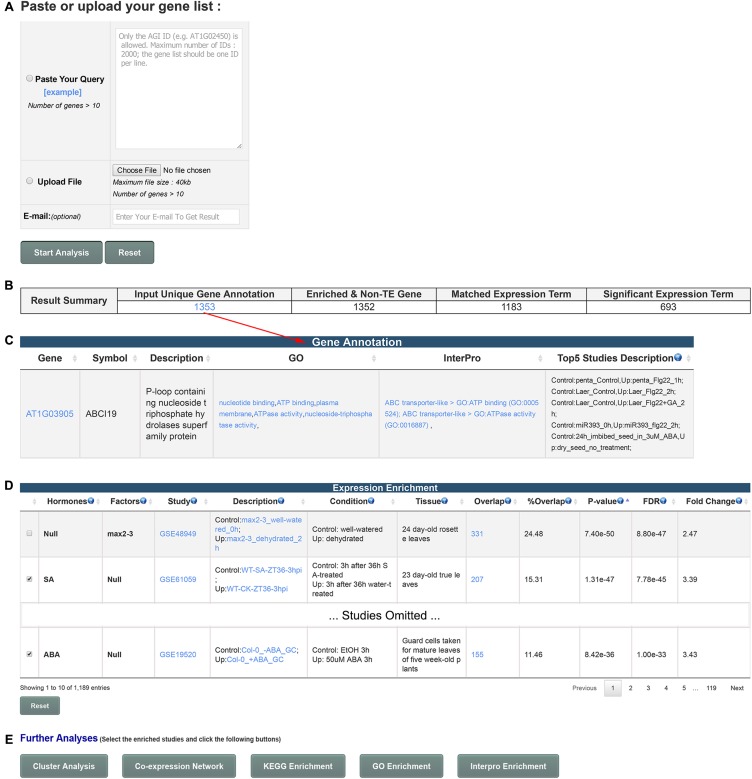
Usage and features of GSHR. **(A)** Input page for submitting an *Arabidopsis* gene list. **(B)** Summary of the number of input genes and matched terms in the database. **(C)** Detailed annotation of input gene list. **(D)** Summary page of the transcriptomic comparisons closely relevant to the input gene list. The hormones or factors associated with the comparisons are listed in the 1st and 2nd columns. **(E)** Optional functional analyses of selected genes from the summary page shown in **(D)** including clustering and visualization of the expression pattern, co-expression network, enrichment analysis of pathways (KEGG), functions (Gene Ontology, GO) and domains (InterPro).

GSHR integrates thousands of hormone-related transcriptomic data sets and offers a user-friendly interface for gene list-level analyses of user input gene lists (**Figure 1**). GSHR accepts gene lists with *Arabidopsis thaliana* Genbank IDs (**Figure 2A**) and returns the hormones and hormone-related factors potentially regulating the input gene list, ranked by statistical significance. Detailed descriptive and statistical information about the comparisons is also presented (**Figures 2B–D**). For those hormones regulated input gene lists, further functional exploration could be directly performed on the resulting page, including hierarchical clustering of expression pattern in response to related hormones, co-expression network, enrichment analyses of pathways, gene ontology terms and InterPro domains. The purpose is to facilitate a more comprehensive understanding about the input gene list, and helping pinpointing essential genes of interest from the input (**Figure 2E** and Supplementary Figure 1). The manual page of the website provides detailed guidelines.

### Examples of Use

#### Case 1: Hormones Involved in Plant Response to Phosphate Deficiency

**FIGURE 3:**
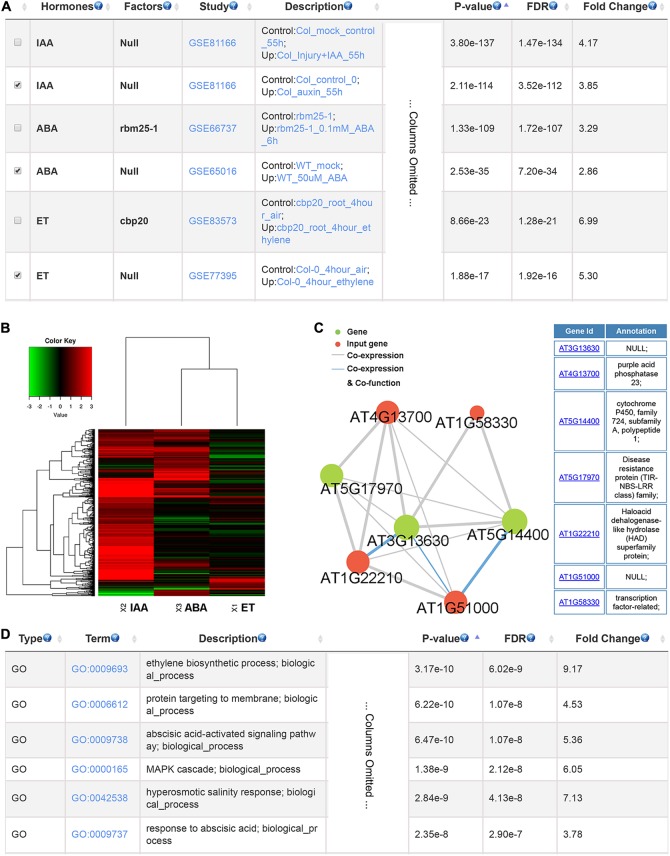
The resulting page summarizing relevant hormones and functional terms enriched for phosphate deficiency-induced genes. The most relevant hormones and factors **(A)**, the clustering and visualization of the expression pattern in response to abscisic acid (ABA) and auxin (IAA) and ethylene (ET) **(B)**, the co-expression network **(C)** and the enriched GO terms **(D)**.

To illustrate the usage of GSHR, we used genes up-regulated by phosphate starvation in *Arabidopsis thaliana* roots as input (Sun et al., 2016). The output given by GSHR showed that a significant proportion of the input genes were regulated by ABA, auxin and ET (**Figure 3A**), indicating that these hormones were potentially involved in plant responses to phosphate deficiency. In support of the results, ABA-responsive genes were induced when cultured in low level of phosphate (Ciereszko and Kleczkowski, 2002; Chiou and Lin, 2011). In addition, low phosphorus enhanced the sensitivity of root response to auxin and ET in *Arabidopsis*, resulting in root architecture changes (Lopez-Bucio et al., 2002; Ma et al., 2003). Following identification of the relevant hormones, the enriched gene sets could be selected for further functional analyses. Clustering and visualization were provided to characterize their expression responses to selected hormones. The heatmap suggested that the selected gene sets were preferentially up-regulated by IAA and ABA, and both up and down-regulated genes were observed by treatment of ET (**Figure 3B**). In addition, genes with high degree in the co-expression network are good candidates of key factors linking signaling pathways between phosphate signaling and hormone responses (**Figure 3C**). Enriched GO terms include ET biosynthesis and ABA signaling pathway as expected (**Figure 3D**).

#### Case 2: Hormones Participated in Plant Response to Cadmium Stress

**FIGURE 4:**
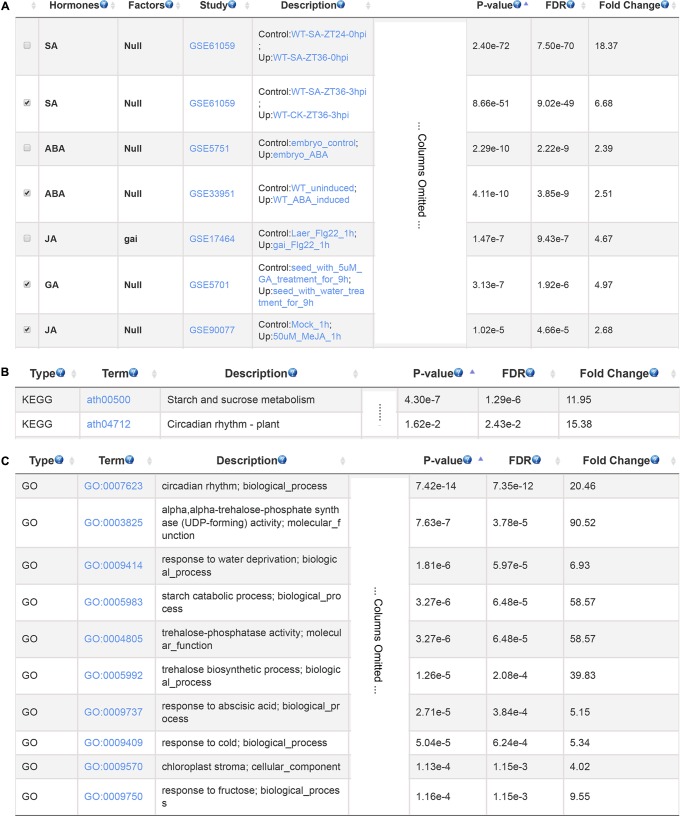
The resulting page summarizing relevant hormones and functional terms enriched for cadmium stress response. The most relevant hormones and factors **(A)**, the enriched pathways **(B)**, and GO terms **(C)**.

Cadmium is a toxic non-biodegradable heavy metal which negatively affect plant growth and development (Bucker-Neto et al., 2017). We used a set of cadmium-induced gene from *Arabidopsis* root as input to search for hormones involved in plant response to cadmium stress (Lopez-Martin et al., 2008). The GSHR identified SA, ABA, GA and JA as top enriched plant hormones (**Figure 4A**). This result is consistent with previous studies. SA influenced the absorption and transport of cadmium and acted as a signaling molecule activating cadmium-tolerant genes (Liu et al., 2016); JA could alleviate negative impacts of cadmium stress by increasing activities of antioxidant enzymes in soybean (Keramat et al., 2009); GA reduced nitric oxide accumulation in roots and suppressed cadmium uptake in *Arabidopsis* to alleviate cadmium toxicity (Zhu et al., 2012); ABA concentration was increased when exposed to cadmium, which induced transient MAP kinase activity thereby increasing cadmium tolerance (Burnett et al., 2000; Bucker-Neto et al., 2017). Further KEGG and GO enrichment results of selected gene sets indicated that these genes mainly related to plant circadian rhythm, starch and sucrose metabolism, ABA and stress response (**Figures 4B,C**), which were also reported to be involved in cadmium responses (Devi et al., 2007; Maistri et al., 2011).

#### Case 3: PIF4 Targets Were Enriched for Auxin- and ABA-Responsive Genes

**FIGURE 5:**
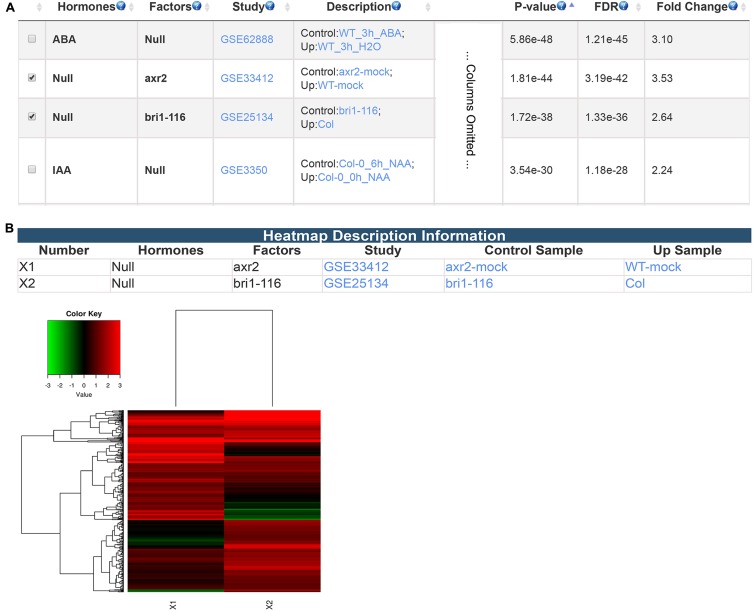
The result pages summarizing the transcriptomic comparisons relevant to PIF4 target genes. The expression enrichment result **(A)** and the clustering analysis of the genes regulated by AXR2 and BRI1 **(B)**.

Besides differentially expressed genes from transcriptomic studies, GSHR accepts gene lists obtained from any source including different types of high-throughput data. Here, PIF4 target genes identified via ChIP-seq experiment (Pedmale et al., 2016) were used as input to illustrate this application of GSHR. AUXIN RESISTANT 2 (AXR2)- and BRASSINOSTEROID INSENSITIVE 1 (BRI1)-regulated genes were among the most enriched gene sets (**Figure 5A**). AXR2 belongs to the Auxin/Indole Acetic Acid protein family essential for mediating auxin signaling (Nagpal et al., 2000) that was also reported to be involved in the BR signaling network (Nemhauser et al., 2004; Nakamura et al., 2006). BRI1 is a leucine-rich repeat receptor kinase involved in BR signal transduction (Wang et al., 2001; Kinoshita et al., 2005). Consistent with the enrichment result, recent genetic and high-throughput studies revealed that PIF4 is closely associated with auxin- and BR-mediated signaling pathways. (Oh et al., 2012, 2014; Wang et al., 2014; Franciosini et al., 2017). PIF4 targets a significant proportion of the genes targeted by AUXIN RESPONSE FACTOR 6 (ARF6) and BRASSINAZOLE-RESISTANT 1 (BZR1), the core mediators of auxin and BR signaling, respectively (Chaiwanon et al., 2016). Input genes could be further clustered according to their expression changes in *arx2* and *bri1* mutants (**Figure 5B**).

## Discussion and Future Plan

GSHR provides data-driven analysis to search for hormones and hormone-related factors affecting transcription of input genes. The purpose of this platform is to link previously generated transcriptomic information to newly generated data, thereby helping to underpin biological insights from omics data and provide clues for subsequent research. This is different from traditional plant hormone databases that have mostly focused on providing comprehensive hormone-related information for individual genes (Peng et al., 2009; Jiang et al., 2011).

Focusing on gene sets facilitates comparison across different platforms and different experiments. However, pair-wise comparison generates multiple similar gene sets resulted from comparisons from similar experiments. The resulting page could be more concise via further clustering or removing redundant results. We choose to provide all pair-wise comparison results because different growth conditions and different way of treatments may affect non-identical gene sets, and all these information may be informative and helpful to users. With the rapid accumulation of high-throughput data, annotations, and references, the gene sets in GSHR will be updated annually.

## Author Contributions

XR and JL constructed the web server. XR, MQ, YW, and JC collected and processed public data. XR and YZ wrote the manuscript.

## Conflict of Interest Statement

The authors declare that the research was conducted in the absence of any commercial or financial relationships that could be construed as a potential conflict of interest. The reviewer BS and handling Editor declared their shared affiliation.

## Footnotes

1 http://www.ncbi.nlm.nih.gov/geo/

2 http://www.bioinformatics.babraham.ac.uk/projects/trim_galore/
