# Supplementary material for: GSHR, a Web-Based Platform Provides Gene Set-Level Analyses of Hormone Responses in Arabidopsis
Source: Front Plant Sci. 2018 Jan 24;9:23. doi: 10.3389/fpls.2018.00023 (PMC5787578; doi:10.3389/fpls.2018.00023)
Supplement: Supplementary file 2 [file Image_1.PDF]

Supplementary Material

GSHR, a Web-based Platform Provides Gene Set-level Analyses of Hormone Responses in *Arabidopsis*

Xiaojuan Ran[1,2], Jian Liu[1,2], Meifang Qi[1,2], Yuejun Wang[1,2], Jingfei Cheng[1,2], Yijing Zhang[1,2*]

*** Correspondence:** Yijing Zhang: zhangyijing@sibs.ac.cn

Supplementary Figures

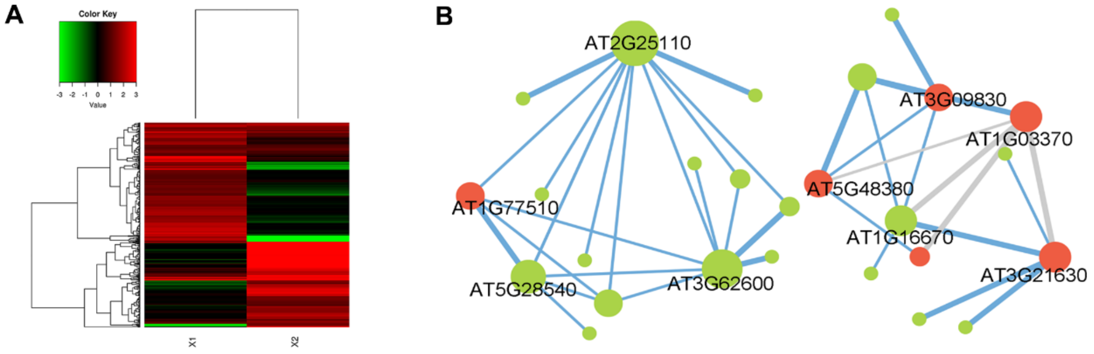

C

KEGG Enrichment

Type	Term	Description	Overlap	%Overlap	P-value	FDR	Fold Change
KEGG	ath04626	Plant-pathogen interaction	15	33	3.54e-10	2.13e-9	9.42
KEGG	ath04016	MAPK signaling pathway - plant	5	11	3.40e-2	9.75e-2	4.04
KEGG	ath00903	Limonene and pinene degradation	3	7	6.00e-2	9.75e-2	7.63

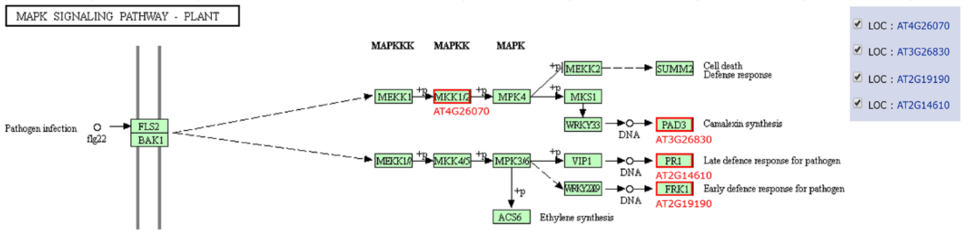

D

GO Enrichment

Type	Term	Description	Overlap	%Overlap	P-value	FDR	Fold Change
GO	GO:0009627	systemic acquired resistance; biological_process	70	24	3.91e-74	7.34e-72	29.37
GO	GO:0009697	salicylic acid biosynthetic process; biological_process	64	22	9.57e-70	8.99e-68	32.35
GO	GO:0009862	systemic acquired resistance, salicylic acid mediated signaling pathway; biological_process	58	20	8.02e-56	5.02e-54	23.15

E

Interpro Enrichment

Type	Term	Description	Overlap	%Overlap	P-value	FDR	Fold Change
Interpro	IPR000719	Protein kinase domain	48	22	9.80e-13	2.35e-11	3.32
Interpro	IPR001611	Leucine-rich repeat	29	13	4.15e-12	4.98e-11	5.31
Interpro	IPR017441	Protein kinase, ATP binding site	35	16	1.65e-10	1.32e-9	3.72

Supplementary Figure 1. The further analyses provided by GSHR. Shown are the result pages of cluster analysis **(A)**, co-expression network **(B)**, enrichment analysis of

pathways **(C)**, GO terms **(D)** and InterPro domains **(E)**.
